# A NMDA-receptor calcium influx assay sensitive to stimulation by glutamate and glycine/D-serine

**DOI:** 10.1038/s41598-017-11947-x

**Published:** 2017-09-14

**Authors:** Hongqiu Guo, L. Miguel Camargo, Fred Yeboah, Mary Ellen Digan, Honglin Niu, Yue Pan, Stephan Reiling, Gilberto Soler-Llavina, Wilhelm A. Weihofen, Hao-Ran Wang, Y. Gopi Shanker, Travis Stams, Anke Bill

**Affiliations:** 1 Novartis Institutes of Biomedical Research, Chemical Biology and Therapeutics, 250 Massachusetts Avenue, Cambridge, MA 02139 USA; 2 Novartis Institutes of Biomedical Research, Global Discovery Chemistry, 181 Massachusetts Avenue, Cambridge, MA 02139 USA; 3 Novartis Institutes of Biomedical Research, Neuroscience, 181 Massachusetts Avenue, Cambridge, MA 02139 USA

## Abstract

N-methyl-D-aspartate-receptors (NMDARs) are ionotropic glutamate receptors that function in synaptic transmission, plasticity and cognition. Malfunction of NMDARs has been implicated in a variety of nervous system disorders, making them attractive therapeutic targets. Overexpression of functional NMDAR in non-neuronal cells results in cell death by excitotoxicity, hindering the development of cell-based assays for NMDAR drug discovery. Here we report a plate-based, high-throughput approach to study NMDAR function. Our assay enables the functional study of NMDARs with different subunit composition after activation by glycine/D-serine or glutamate and hence presents the first plate-based, high throughput assay that allows for the measurement of NMDAR function in glycine/D-serine and/or glutamate sensitive modes. This allows to investigate the effect of small molecule modulators on the activation of NMDARs at different concentrations or combinations of the co-ligands. The reported assay system faithfully replicates the pharmacology of the receptor in response to known agonists, antagonists, positive and negative allosteric modulators, as well as the receptor’s sensitivity to magnesium and zinc. We believe that the ability to study the biology of NMDARs rapidly and in large scale screens will enable the identification of novel therapeutics whose discovery has otherwise been hindered by the limitations of existing cell based approaches.

## Introduction

N-methyl-D-aspartate-receptors (NMDARs) are ionotropic glutamate receptors which require binding of two different ligands, glutamate and either glycine or D-serine for their activity. NMDARs have been studied extensively in the context of neuroscience due to their involvement in synaptic transmission, plasticity, cognition and disease. Genetic and functional studies have implicated NMDARs in schizophrenia and other nervous system disorders such as epilepsy, stroke, pain, addiction, depression and Alzheimer’s disease^1, 2^. In addition to CNS disorders, the presence and/or the effect of these receptors in other organ systems have led to suggest NMDARs as targets for diseases such as diabetes^3^, inflammatory bowel syndrome^4^, glaucoma^5^ and in immune dysfunction^6, 7^. Therefore, the ability to identify small molecules that modulate NMDAR function is of high interest.

Studying NMDARs in cell based systems is challenging. The receptor architecture is complex, composed of at least two out of seven different subunits, which confer the receptor with distinctive properties^8^. NMDARs are heterotetramers, consisting of two obligatory GluN1 (NR1) subunits, which bind glycine or D-serine, combined with two subunits of GluN2 (NR2A, NR2B, NR2C, NR2D) and/or two GluN3 (NR3A and NR3B) subunits that all bind glutamate. These can form either di-heteromeric (e.g. two NR1 and NR2A subunits, respectively) or more complex tri-heteromeric (e.g. two NR1 subunits, one NR2A and one NR2B) receptor complexes^2, 8^. Functional activation of NMDARs requires binding of both ligands, glycine/D-serine and glutamate, and membrane depolarization, which removes a magnesium ion from its binding site within the ion conduction pore. Hence, NMDARs act as coincident detectors, coordinating presynaptic neuronal activity with postsynaptic depolarization. This unique ability to integrate pre- and post-synaptic signals make NMDARs key mediators of synaptic plasticity, a process by which the efficacy of synapses changes over time as result of neuronal activity^9, 10^. NMDAR activation alters the balance of postsynaptic calcium and consequently triggers a cascade of downstream signaling events affecting the activity, expression and/or localization of various mediators of postsynaptic signaling, including NMDAR itself, thereby enhancing or weakening synaptic strength^11^. Because distinct biophysical properties and expression patterns of NMDARs containing different NR2 subunits are likely to play specific roles in synaptic plasticity and disease^12^, identifying subunit-selective modulators may offer the potential to engage more specific neuronal processes as well as mitigate potential side effects caused by general modulation of NMDAR activity.

One of the greatest challenges in studying these receptors in cell-based, HT (high throughput) platforms is that overexpression of functional NMDARs in non-neuronal cells result in cell death due to constitutive activation of the receptor at depolarized membrane potential^13^. Different approaches have been developed to study NMDARs, mainly using stable cell lines that overexpress different combinations of receptor subunits^14–17^. The most common approach to study NMDARs in cells is via electrophysiological measurements such as patch clamping. Although these methods provide a pleiotropy of different data readouts, throughput is limited and the costs per sample usually are prohibitive of larger sample numbers. For larger throughput, measurement of calcium influx using fluorescent dyes has been widely used as a method to identify modulators of NMDAR activity in a microplate-based format. To limit cell toxicity in these systems, cells are typically engineered to express only one subunit constitutively (e.g. NR2A) whilst the other (e.g. NR1) subunit is expressed under the control of an inducible promotor, e.g. tetracycline induced expression. However, even in such systems, due to the high expression levels after induction, the presence of functional receptors is highly toxic, requiring cell cultures to be maintained in the presence of potent channel blockers such as Ketamine^14, 15^. However, these channel blockers are hard to wash-out and toxic to the cells resulting in cell death and release of glycine and glutamate, which occupy the ligand binding sites and occlude the pharmacological modulation of receptor activity. Channel blockers may therefore confound experiments that measure calcium signaling in a high-throughput fashion. To circumvent this problem other groups have included NMDA-receptor antagonists in their experiments to preserve the inactive form of the receptor. However, high concentrations of ligands are required to compete with the antagonists present during the assay, aggravating the identification of NMDAR modulators^16, 17^. In addition to already outlined complications, NMDAR expressed in these assays is no longer sensitive to glycine/D-serine stimulation, preventing the investigation of D-serine/glycine-dependent mechanisms^18^.

Here we report a novel, plate-based, high-throughput approach to study NMDARs that overcomes all the limitations of available approaches. The method allows for rapid assessment of functional NMDARs with different subunit composition and of agonist effects for both, the glycine/D-serine as well as the glutamate binding site. The method uses baculovirus^19^ as vectors to introduce different receptor subunits or receptor mutants in a flexible and facile way, enabling the study of subunits including their specific pharmacological modulation. As opposed to channel blockers like Ketamine or MK-801, our approach leverages the use of weak glycine and glutamate binding site antagonists, to mitigate cellular toxicity and to allow access to ligand-free states of NMDARs. The selected weak antagonists can be readily washed off, so that the freed up binding sites allow NMDAR activation by D-serine/glycine and glutamate binding. This allows to measure the ligand-induced activity of NMDAR via calcium imaging and to investigate the effect of small molecule modulators on the activation of NMDARs at different concentrations or combinations of the co-ligands. The reported assay system faithfully replicates the pharmacology of the receptor in response to known agonists, antagonists, positive and negative allosteric modulators, as well as sensitivity to magnesium and zinc. We believe that the ability to study the biology of NMDARs rapidly and in large scale will enable the identification of novel therapeutics whose discovery has otherwise been hindered by the limitations of existing cell based approaches.

## Results

### Rapid, titratable expression of NMDAR in HEK293 cells using baculovirus enables optimization of NMDAR functional expression levels

**Figure 1:**
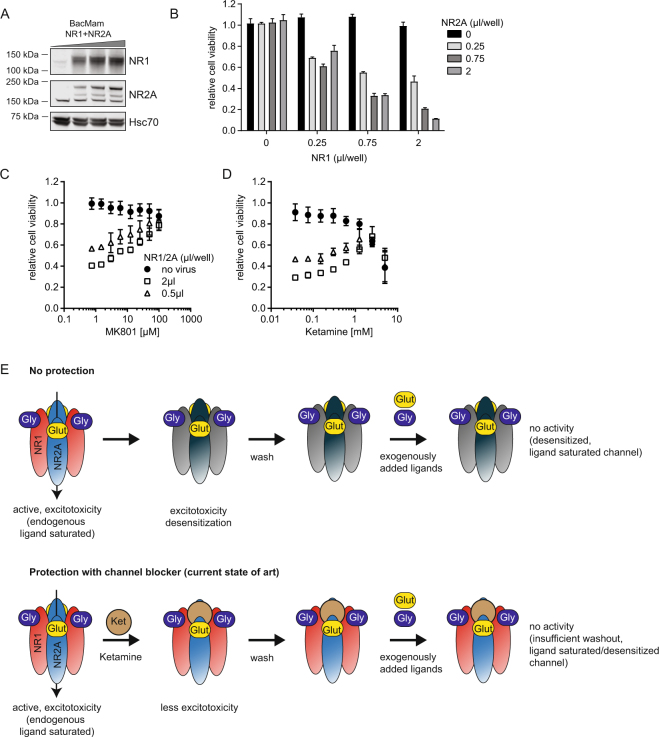
Functional expression of NMDAR in HEK293 cells using baculovirus. **(A)** NR1 and NR2A protein levels in HEK293 cells transduced with baculovirus. HEK293 cells were transduced in the presence of 1 mM Ketamine with different amounts of baculovirus encoding human NR1 and NR2A. Cells were harvested 16 h after transduction, and NR1 and NR2A protein levels were analyzed by immunoblotting. Representative images are shown. Molecular weight standards are indicated. NR2A shows an unspecific band at ~150 kDa. **(B)** Cell viability of HEK293 cells 16 h after transduction with different amounts of baculovirus encoding NR1 or NR2A. Cell viability was assessed using CellTiterGlo reagent. Data was normalized to non-transduced cells and represents the mean ± standard error of the mean (SEM) of three experiments. **(C,D)** Cell viability of HEK293 cells 16 h after transduction with baculovirus encoding NR1 or NR2A with concurrent treatment with MK801 (**C**) or Ketamine (**D**). Data was normalized to non-transduced cells in the presence of DMSO and represents the mean ± SEM of three experiments. **(E)** Model for lack of NMDAR activity in functional assay after protection with MK801 or Ketamine. NMDAR-ligands contained or secreted by the cells into the media activate NMDAR and lead to excitotoxicity and desensitization of the channel. Protection with MK801 or Ketamine prevent excitotoxicity, however, both compounds are difficult to wash out and do not prevent occupation of the ligand binding sites with endogenous ligand and prevent NMDAR activity in functional assays.

Ectopic expression of functional NMDAR in non-neuronal cells is known to result in significant excitotoxicity and cell death^13^. The toxic effect correlates with NMDAR expression levels. NMDAR inhibitors can protect from cytotoxicity but they can interfere with NMDAR activity in functional downstream assays. Hence, high expression levels of NMDAR, that allow for a good assay signal window need to be balanced with minimum cell death and the amount of protective NMDAR-inhibitors needed. Here, we developed a baculovirus based NMDAR expression system to achieve rapid, titratable and optimal expression levels of individual NMDAR subunits in non-neuronal cells. Transduction of HEK293 cells with baculovirus encoding for NR1 and NR2A in the presence of Ketamine resulted in expression of both NMDAR subunits after only 16 h as assessed by immunoblotting (Fig. 1A). NMDAR expression levels correlated with the amount of virus added, demonstrating the advantage of baculovirus to enable titratable expression levels of NMDAR simply by adjusting the amount of virus added to the cells. Titration experiments of baculovirus encoding NR1-/NR2A-subunits of NMDAR showed that cell viability was dependent on the expression levels of both subunits (Fig. 1B). NMDAR-mediated cytotoxicity could be rescued by addition of the channel blockers MK801 or Ketamine in a concentration dependent manner (Fig. 1C,D). Lower virus amounts and hence lower NMDAR expression levels required lower concentrations of inhibitor to maintain cell viability (Fig. 1E). Ketamine itself showed a dose-dependent toxic effect on HEK293 cells (Fig. 1D), likely causing cell stress and/or toxicity which might interfere with downstream experiments.

### Agents protecting from excitotoxicity are mostly incompatible with plate based readouts of NMDAR activity

**Figure 2:**
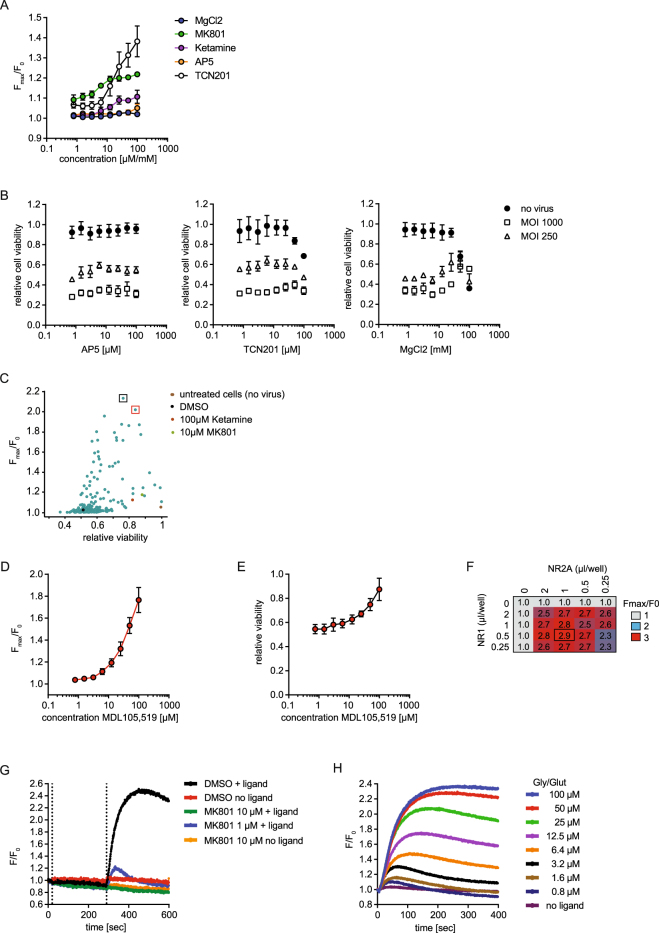
Protection of NMDAR-expressing cells with MDL105,519, but not MK801 or Ketamine, facilitates NMDAR-activity in functional assay. **(A)** NMDAR-mediated calcium flux in HEK293 cells transduced with baculovirus. NR1/2A-transduced HEK293 cells were treated with the indicated compounds. 16 h later, cells were washed three times, loaded with Calcium6 dye and NMDAR-mediated calcium flux was measured after stimulation with 100 µM glycine/glutamate. Data represents the mean ± SEM of the maximal fluorescence ratio (maximal fluorescence/baseline fluorescence, Fmax/F0) of three experiments. Units for x-axes are mM for MgCl_2_ and µM for all other inhibitors. The raw traces for a representative experiment are shown in Supplementary Figure 1. **(B)** Cell viability of NR1/2A-transduced cells with concurrent treatment with the indicated NMDAR-inhibitors was measured 16 h after infection using CellTiterGlo. Data was normalized to non-transduced cells in the presence of DMSO and represents the mean ± SEM of three experiments. **(C)** Identification of NMDAR protective compounds. Cells were transduced with baculovirus encoding NR1/NR2A with concurrent treatment with ~120 NMDAR-inhibitors (3 µM or 100 µM), and analyzed as in (**A** and **B**). Data represents the mean of two replicates and was normalized to DMSO cells (no virus). The black and red box highlights the two best screening hits: 100 µM MDL105,519 and 3 µM CGP070667, respectively. **(D)** NMDAR-mediated calcium flux in presence of MDL105,519. Cells were prepared as in (**A**) and treated with different concentrations of MDL105,519. Data represents the maximal fluorescence ratio, ± SEM, four experiments. **(E)** Cell viability of NMDAR-expressing cells in presence of MDL105,519. Cells were prepared as in (**D**) and analyzed as in. (**B**) Data represents the mean ± SEM of four experiments. **(F)** Titration of the maximal NMDAR-mediated calcium flux in HEK293 cells transduced with baculovirus. Cells were prepared and analyzed as in (**D**). Data represents the mean of the maximal fluorescence ratio of three independent wells. **(G)** Test for ligand-independent activity of NMDAR. Cells were prepared as in (**D**). MK801 or DMSO was added and NMDAR-mediated calcium flux was measured for 5 min before and after addition of 100 µM glutamate/glycine. Data represent the fluorescence ratio shown as raw data of representative wells. **(H)** Ligand concentration dependency of NMDAR-mediated calcium flux. Cells were prepared as in (**D**). Data represent the fluorescence ratio shown as raw traces of representative wells.

Next we asked whether MK801 or Ketamine protected NMDAR-expressing cells can be used to measure ligand-induced NMDAR activity in a calcium flux assay. We did not observe significant ligand-induced NMDAR-mediated calcium influx in the cells, even after multiple wash cycles to remove the compounds (Fig. 2A, Supplementary Figure 1). This observation indicates that MK801- and Ketamine-mediated protection from excitotoxicity is not suitable to enable the measurement of NMDAR activity in a microplate based assay, likely because of the inability to completely wash out the inhibitors (Fig. 1E). Next we asked if NMDAR channel blockers can be substituted with compounds that feature alternative modes of inhibition, e.g. the allosteric negative modulator TCN201^20^, the NR2A ligand-binding site antagonist AP5^21^ or pore blocking MgCl_2_^8^. To test this, we treated transduced cells with the indicated inhibitors and measured calcium influx and cell viability. Neither AP5 nor TCN201 protected the cells from excitotoxicity, while MgCl_2_ showed partial rescue at high concentrations (>10 mM, Fig. 2B). None of the tested compounds facilitated a ligand-induced NMDAR-mediated calcium influx in our plate based measurement. Taken together, these data indicate that protection from excitotoxicity is not sufficient to enable the measurement of NMDAR-dependent calcium signals in micro-plate based assays with limited washing capability.

### The antagonist MDL105,519 alleviates cytotoxicity and enables ligand-induced NMDAR activity

The lack of ligand-induced NMDAR activity after culturing the cells in the presence of NMDAR-channel blockers highlights the need to find protective compounds with more favorable bio- and physicochemical properties to facilitate their removal before NMDAR activation can be measured. Hence, we profiled an informed set of 120 compounds consisting of known NMDAR-inhibitors and their analogs for their cell-protective effects and ease of wash out. Cells transduced with NR1/2A baculovirus were treated overnight with compound, followed by washout of the compounds and measurement of both glutamate and D-serine induced calcium influx as well as cell viability. As shown in Fig. 2C, we identified multiple compounds that significantly improved cell viability and facilitated the measurement of calcium influx. Treatment with the NR1A glycine-competitive antagonist MDL105,519 (2,3-dioxo-1,4-dihydroquinoxalin-5-yl)methylphosphonic acid^22^ enabled the largest dynamic range of NMDAR-activity and maintained cell viability at >70% compared to non-transduced cells. Treatment of NR1/2A transduced cells with MDL105,519 resulted in a dose-dependent rescue of cell viability and facilitated a significant and stable signal in the calcium flux assay (Fig. 2D,E). We chose 100 µM MDL105,519 as the optimal concentration for further experiments, and re-optimized the amount of NR1 and NR2A baculovirus needed to achieve optimal functional expression levels in presence of MDL105,519 for subsequent NMDAR-mediated calcium flux assays (Fig. 2F). A 1:2 (vol/vol) ratio of NR1/NR2A virus was found to be optimal and used in subsequent experiments. At this expression level of NMDAR, receptor activity in the absence of ligand was insignificant (Fig. 2G). NMDAR block by MK801 revealed only a weak decrease of signal in the absence of ligand. This suggests a low basal activity of NR1/2A after washout of MDL105,519 (Fig. 2G), which is much lower compared to the activity in alternative expression systems reported for NMDAR^17^. Therefore, tuned expression of NMDAR with improved cell viability and low activation levels, triggered by endogenous ligands, are clear benefits of our method. Next, we stimulated NR1/2A-transduced and MDL105,519 protected HEK cells, after washout of the antagonist, with varying concentrations of glycine and glutamate and showed a ligand-concentration dependent activity of NMDAR as measured by NMDAR-mediated calcium influx (Fig. 2H). Taken together, our results demonstrate that baculovirus mediated expression of NMDAR in HEK293 cells in the presence of MDL105,519 enables ligand-dependent NMDAR activity in non-neuronal cells without having a protecting compound present during the measurement.

### Enabling sensitivity of NMDAR to glycine and glutamate (in a plate based assay)

**Figure 3:**
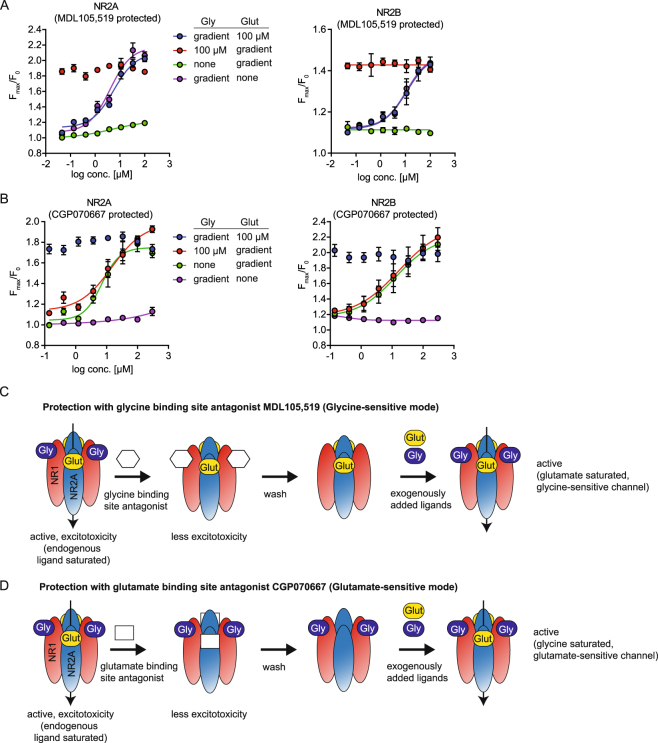
Baculovirus mediated expression of NMDAR facilitates sensitivity of NMDAR to ligands of both the glycine- and the glutamate binding sites. **(A)** Glycine sensitivity of NMDAR-mediated calcium flux in MDL105,519 protected cells. HEK293 cells were transduced with NR1/2 A or NR1/2B baculovirus in the presence of MDL105,519 and NMDAR-mediated calcium flux was measured as described after addition of the indicated concentration of glycine and glutamate. Data represents the mean of the maximal fluorescence ratio ± SEM of three experiments. **(B)** Glutamate sensitivity of NMDAR-mediated calcium flux in CGP070667 protected cells. HEK293 cells were transduced with NR1/2 A or NR1/2B baculovirus in the presence of CGP070667 and NMDAR-mediated calcium flux was analyzed as in (**A**). **(C,D)** Model of protection facilitated sensitivity of NMDAR activity to glycine (**C**) and glutamate (**D**). Protection of NMDAR by addition of ligand binding site antagonists protect NMDAR by occupying the ligand binding site and prevent binding of endogenous ligands. After washout of the antagonist, the ligand binding sites become accessible for exogenously added ligand, hence facilitating ligand-sensitivity.

Intrigued by the finding that protection of NMDAR-expressing cells from excitotoxicity using an antagonist of the glycine binding site facilitated NMDAR function, we reasoned that MDL105,519, when washed out, would render NMDAR sensitive to exogenously added glycine. With this cell-conditioning procedure, the antagonist would prevent the endogenous ligand from binding to the receptor and would prevent activation and subsequent desensitization of the channel. Once the antagonist is washed out, the channel remains in its ligand-free form and would hence be sensitive to the addition of glycine. To test this hypothesis, we transduced HEK293 cells with NR1/2A and protected the cells with MDL105,519. After wash out of the compounds, NMDAR-mediated calcium flux in presence of varying concentrations of glycine, glutamate alone or combinations of both ligands was measured. As shown in Fig. 3A, MDL105,519-protected cells showed a dose-dependent activation by glycine and D-serine with an EC_50_ of ~12 µM and ~9 µM, respectively (Supplementary Figure 2A). L-serine showed a ~200-fold right shift in potency as compared to D-serine likely caused by contamination of the commercially available L-serine reagents with traces of D-serine (Supplementary Figure 2A). No significant dose-dependent activation by glutamate was detected. These data support our model, in which the antagonists protect the ligand binding site from endogenous ligand present in the cell media and, once washed out, increase the fraction of ligand-free receptor on the cell membrane which can subsequently be activated by the addition of exogenous ligand (Fig. 3C,D). Analogously, a glutamate binding site antagonist would facilitate glutamate sensitivity of the channel. From our set of 120 compounds profiled for NMDAR protection (Fig. 2C), we identified CGP070667^23^ as a glutamate competitive antagonist providing the best protection from NMDAR-mediated excitotoxicity. Protection of the cells with CGP070667 and subsequent washout resulted in a dose-dependent sensitivity to glutamate (EC_50_~10 µM), but insensitivity to glycine (Fig. 3B). An excess concentration of 100 µM glycine further increased the signal, but did not affect the EC_50_ of glutamate, indicating that the endogenous ligand concentration is insufficient to saturate the glycine binding site of the receptor. To our knowledge, we have developed the first cellular, plate-based assay that enables the study of both, glycine/D-serine and glutamate dependent NMDAR activity. Our approach is not limited to baculovirus mediated expression of NMDAR, but can be used with cell models that stably express NMDAR under the control of an inducible promotor (Supplementary Figure 2B) and that lacked sensitivity to activation by glycine/D-Serine^18^, highlighting the wider applicability of this approach.

### NR1/2B sensitivity to glycine and glutamate can be enhanced in a similar fashion

Next we asked whether our approach to use weak antagonist to protect the ligand binding site from endogenous ligand could be used to study NMDARs with other subunit compositions. To optimize functional expression levels for NR1/2B we followed the protocol developed for NR1/NR2A and obtained an optimal ratio of 1:4 NR1/NR2B (vol/vol). Similarly to NR1/2 A, NR1/2B-mediated calcium flux was sensitive to stimulation with glutamate and glycine in a concentration dependent manner (Fig. 3A,B). NR1/2B showed overall weaker signal and slower kinetics compared to NR1/2 A as previously observed in electrophysiological assays^8^. Taken together, these data show that our approach can be applied to other NMDAR subunits, further highlighting the flexibility and advantage of baculovirus-mediated expression of NMDARs and establishing a new assay to study NR1/2B activity in non-neuronal cells.

### Membrane potential-dependent activity and pharmacological modulation of NMDAR

Next we asked, whether our approach faithfully replicates expected NMDAR biology and pharmacology.

**Figure 4:**
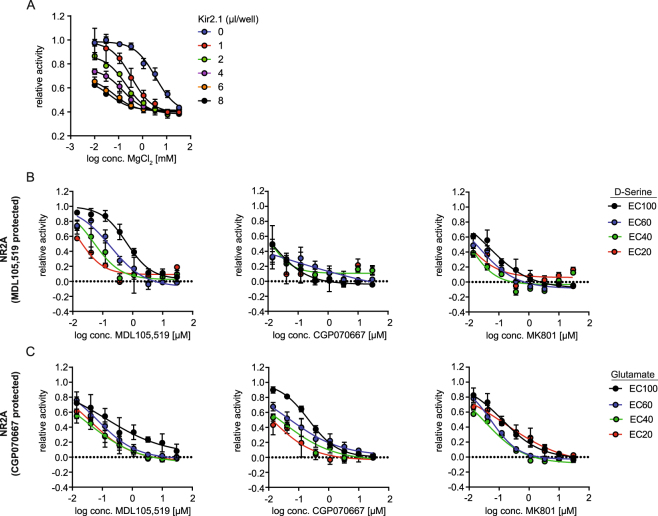
Characterization of the mode of action of NMDAR inhibitors. **(A)** Sensitivity of NMDAR-mediated calcium flux to inhibition by magnesium at varying membrane potentials. HEK293 cells were transduced with baculovirus encoding KCNJ2 (Kir2.1), NR1 and NR2A in the presence of MDL105,519 and NMDAR-mediated calcium flux was measured as previously described in the presence of varying amounts of magnesium chloride. The maximal fluorescence ratio was normalized to NR1/2A-transduced cells in the absence of magnesium. Data represents the mean ± SEM of three experiments. **(B,C)** Sensitivity of NR1/2 A mediated calcium flux to inhibition by MDL105,519 (first column), CGP070667 (middle column) or MK801 (last column) at different ligand concentrations. HEK293 cells were transduced with baculovirus encoding NR1 and NR2A in the presence of MDL105,519 (**B**) or CGP070667 (**C**) and NMDAR-mediated calcium flux was measured as described in the presence of the indicated ligand concentrations. The integral of the maximal fluorescence ratio was normalized to the activity measured in the absence of inhibitor (DMSO only) at the respective ligand concentration. Data represents the mean ± SEM of three experiments.

One hallmark of the NMDAR is the strong voltage-dependence of magnesium block, which controls channel activity^2, 8^. HEK293 cells are known to have a depolarized resting membrane potential as compared to neuronal cells^24^ (we determined a resting membrane potential of ~−30 mV for HEK293 cells; data not shown), hence magnesium is not expected to contribute to NMDAR regulation in our assay conditions. Hyperpolarization of the membrane potential in HEK293 cells is expected to result in an increased sensitivity of NMDAR to inhibition by magnesium. In order to modify the resting membrane potential in NR1/2A-transduced HEK293 cells and to study its effects on the sensitivity of NMDAR to inhibition by magnesium, we co-transduced the cells with baculovirus encoding the inward rectifying potassium ion channel Kir2.1 (KCNJ2)^24^. Figure 4A shows that increasing Kir2.1 baculovirus titer resulted in increased sensitivity of NR1/NR2A to inhibition by magnesium, consistent with the expected behavior of NMDAR from electrophysiology experiments in neurons^2, 8^. To our knowledge, our data introduces the first facile and rapid, non-electrophysiology based model to study the membrane potential-dependent effect of magnesium on NMDAR activity in non-neuronal cells.

To further validate the utility of our assay, we profiled several known NMDAR antagonists and allosteric modulators to test whether known pharmacology could be reproduced in our assay. NMDAR-mediated calcium flux of NR1/2A- or NR1/2B-transduced and MDL105,159 protected HEK293 cells was measured in the presence of varying concentrations of NMDAR inhibitors. Table 1 lists all compounds tested, their described mechanisms of action as well as the IC_50_ values obtained in our system. Taken together, the data shows that our system reproduced expected NMDAR pharmacology and the assay system is both biologically and pharmacologically relevant.

**Table 1:** Measured IC50s of NMDAR inhibitors.

Compound	MoA	Reference	NR2A	NR2B
TCN201	negative allosteric modulator, NR2A selective	20	11.93	n.d.
Ifenprodil	negative allosteric modulator, NR2B selective	47	n.d.	0.10
CP101606	negative allosteric modulator, NR2B selective	48	n.d.	0.02
RO25-6981	negative allosteric modulator, NR2B selective	49	n.d.	0.10
7-CTKA	glycine site antagonist	51	28.02	28.32
MDL105,519	glycine site antagonist	22	0.62	1.65
L701,324	glycine site antagonist	58	1.45	1.91
CGP070667	glutamate site antagonist	23	0.07	0.37
NVP-AAM077	glutamate site antagonist	54	2.46	11.86
MK801	channel blocker	50	0.10	0.15
Ketamine	channel blocker	59	23.00	12.14
QNZ46	NR2C/2D selective antagonist	55	n.d.	n.d.

Data represents the mean of the IC50 (µM) as determined in NR1/2A transduced HEK293 cells after protection with MDL105,519 in n > 3 experiments after stimulation with EC80 of both ligands. n.d.: not detectable

### Dual ligand sensitivity of NMDAR enables dissection of the mechanism of action of NMDAR inhibitors

Next we set out to use our system to determine the mechanism of action of NMDAR inhibitors. We hypothesized that exogenously added D-serine, but not glutamate, would compete with the effect of a D-serine binding site antagonist and would result in a right shift of the dose response curve. Analogously, addition of glutamate, but not glycine, would right shift the dose response curve of a glutamate binding site antagonist. Neither excess of D-serine nor glutamate would show an effect on the potency of a channel blocker. To test this, NR1/2A-transduced HEK293 cells were protected with either MDL105,519 or CGP070667 and NMDAR-mediated calcium flux was measured after stimulation with EC_20_/_40_/_60_/_100_ of either D-serine or glutamate in the presence of varying concentrations of NMDAR-inhibitors. Stimulation with the EC_100_ of D-serine caused a right shift of the dose response curve for MDL105,519 as compared to the EC_20_ of D-serine, while there was little effect on the potency of CGP070667 or MK801 (Fig. 4B). Similarly, CGP070667 showed a glutamate-dependent shift in potency, while the concentration of glutamate had only minor effects on the potency of MDL105,519 or MK801 (Fig. 4C). Similar results were obtained for NR1/2B (Supplementary Figure 3A,B). This data is consistent with the hypothesized behaviors of NMDAR-antagonists and channel blockers. Taken together, our results demonstrate the ability of our assay to dissect the mechanism of action of NMDAR inhibitors, enabling studies to determine the mode of action of novel compounds and the design of mechanism-specific compound screens.

### Studying the mode of action of positive allosteric modulators and activators of NMDAR

Next we used our sytem to characterize two bona fide positive allosteric modulators (PAMs) of NMDAR. For this, we measured the effect of the PAMs at the EC30 of either D-serine or glutamate, as well as in the presence of saturating amounts of both ligands. This allowed us to assess the ligand-dependent as well as the ligand-independent effect of the PAMs, respectively.

**Figure 5:**
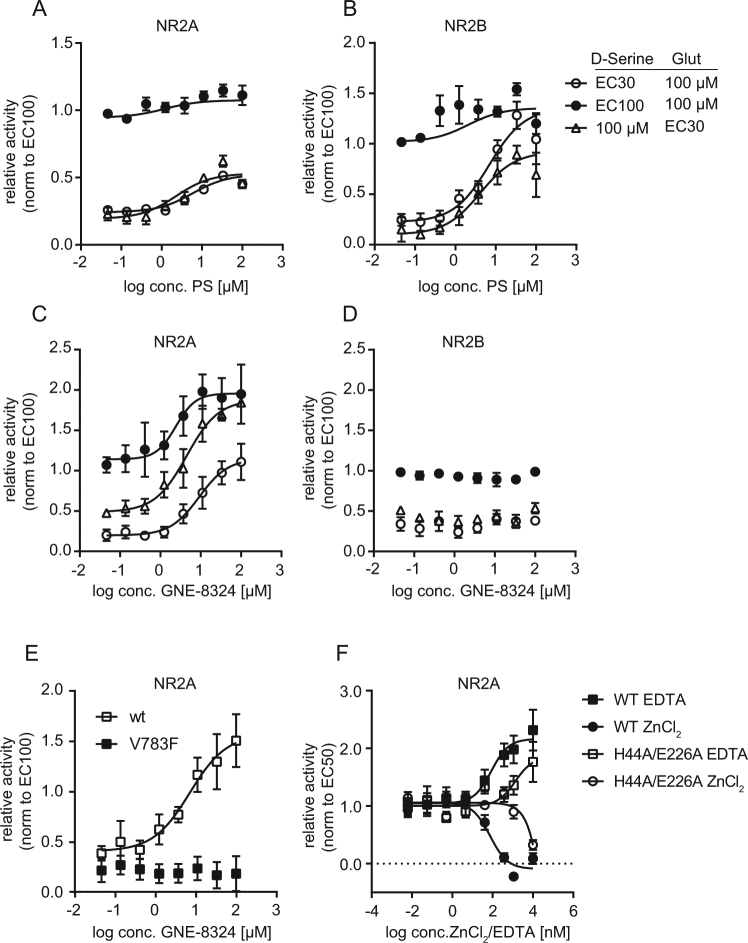
Characterization of the mode of action of positive allosteric modulators and activators of NMDAR **(A,D)** Effect of positive allosteric modulators of NMDAR on NR1/2A- or NR1/2B-mediated calcium flux. HEK293 cells were transduced with baculovirus encoding NR1 and NR2A or NR2B in the presence of MDL105,519 or CGP070667, and NMDAR-mediated calcium flux was measured as described in the presence of the indicated ligand concentrations. Cells were incubated with the indicated concentrations pregnenolone sulfate (PS) (**A**,**B**) or GNE-8324 (**C**,**D**) for 5 min before addition of ligand. The data was normalized to the activity in the presence of saturating amounts of both ligands in the presence of DMSO. Data represents the mean ± SEM of three experiments. **(E)** Sensitivity of NR1/NR2A-V783F to GNE-8324. HEK293 cells were transduced with baculovirus encoding NR1 and NR2A-V783F in the presence of MDL105,519 and NMDAR-mediated calcium flux was measured as previously described in the presence of the indicated concentrations of GNE-8324. The data was normalized to the activity in the presence of saturating amounts of both ligands in the presence of DMSO. Data represents the mean ± SEM of three experiments. **(F)** Sensitivity of NR1/NR2A-H44A/E226A to zinc and EDTA. HEK293 cells were transduced with baculovirus encoding for NR1 and either wildtype NR2A or NR2A-H44A/E226A in the presence of MDL105,519, and NMDAR-mediated calcium flux was measured at the EC50 of D-serine as previously described in the presence of the indicated concentrations of zinc or EDTA. The data was normalized to the activity of a DMSO-treated control. Data represents the mean ± SEM of three experiments.

First, we tested pregnenolone sulfate (PS), a naturally occurring neurosteroid that was found to cause potentiation of NMDAR by increasing channel open probability by a yet unknown mechanism^25^. PS enhanced NMDAR activity in the presence of submaximal concentrations of D-serine and glutamate in NR1/NR2A- and NR1/NR2B-transduced HEK239 cells (Fig. 5A,B). PS caused supramaximal activation of NMDAR in the presence of saturating concentrations of ligand (Fig. 5A,B), suggestion a ligand-independent mechanism of action, consistent with the proposed mechanism of action of PS to increase channel open probability^25^.

Next, we characterized the effect of the recently published NR2A-specific PAM GNE-8324^26^. GNE-8324 caused a concentration-dependent potentiation of NR1/NR2A activity at submaximal ligand concentration that was especially pronounced in the presence of limiting amount of glutamate (Fig. 5C) and that significantly exceeded the NMDAR activity observed in the presence of saturating concentrations of both ligands, therefore demonstrating supramaximal activation of the receptor. Concordantly, GNE-8324 also enhanced NMDAR activity at saturating concentrations of both ligands (Fig. 5C). These data indicate a ligand-independent activation component for GNE-8324, e.g. by slowing down channel deactivation. Interestingly, GNE-8324 was not able to cause a supramaximal activation of NR1/2A when glutamate levels were saturating and D-serine levels were limiting, suggesting an additional glutamate-dependent mechanism of action, e.g. increasing the sensitivity to glutamate. GNE-8324 had no effect on NR1/2B (Fig. 5D). Taken together, this data is consistent with the described mode of action of GNE-8324, i.e. potentiation of NR1/2A-activity by increasing sensitivity to glutamate and decreasing channel deactivation kinetics^26^. The data also demonstrates the ability of our assay to identify positive allosteric modulators of NMDAR and to provide important insights into their mechanism of action.

### NMDAR-mutants allow mechanistic studies of receptor function

While previously described assays require the generation of stable cell lines and possible clonal selection, baculovirus-mediated expression of NMDAR offers a flexible and time-saving route to characterize NMDAR mutants. To demonstrate this advantage, we generated baculovirus for two NR2A mutants : NR2A-V783F, a mutant described to block binding of GNE-8324^26^ and double mutant NR2A-H44A/E226A^27^, with alanine mutations of the two residues that coordinate zinc in the amino-terminal domain of NMDAR. Both mutants were co-expressed with wildtype NR1 and showed similar expression levels and sensitivity to stimulation with glutamate (data not shown) as the wildtype NR2A subunit. HEK293-cells transduced with NR1/2A-V783F showed a complete loss of sensitivity to GNE-8324 in the calcium flux assay (Fig. 5E), as previously described in stable cell lines^26^. Transduction of HEK293 cells with NR1/NR2A-H44A/E226A resulted in strong desensitization to zinc inhibition with an IC50 for zinc of >10 µM, compared to the wildtype receptor with an IC50 of ~100 nM. Furthermore, the double mutant was less sensitive to activation by EDTA, likely due to chelation of residual zinc in the assay buffer (Fig. 5F). This result is consistent with the expected compromised ability to bind zinc of this mutant and introduces a novel way to study the zinc-dependent mechanism of NMDAR regulation and modulation.

Taken together, the data presented here introduce a novel dual-ligand sensitive model system for studying NMDAR biology and modulation, including the pharmacological characterization of NMDAR inhibitors as well as positive allosteric modulators. Our assay overcomes the limitations of existing cell based approaches and enables the identification of novel therapeutics for various NMDAR-dependent pathologies.

## Discussion

In this work, we report an assay based on baculovirus-mediated expression of NMDAR to study the pharmacology of NMDARs. We take advantage of weak antagonists that protect cells from NMDAR-induced excitotoxicity, that are easily removed before the measurement and thereby confer sensitivity to stimulation of NMDAR activity by both glutamate and D-serine/glycine. This approach overcomes many of the limitations of currently available assays that monitor NMDAR function in a plate based format and is exceptionally suited to identify NMDAR modulators, i.e. inhibitors and activator, in a high throughput fashion.

Many pharmacological and receptor characterization studies to date are performed in medium throughput, automated electrophysiological platforms or in low throughput manual patch clamp assays, coming with the limiting factors of high costs, limited success rates and long measurement times. Current plate-based approaches on the other hand are confounded by the high background levels of endogenous glutamate and glycine/D-serine. High expression levels of functional NMDAR, as found in stably transfected cell lines, are toxic for the cells because dying cells release glutamate and glycine/D-serine into the media, which subsequently activate NMDARs and promote excitotoxicity. Hence, high concentrations of NMDAR inhibitors are required to protect the cells from excitotoxicity which compromise the ability to detect functional responses mediated by NMDAR activity^16, 17^. In contrast, our assay uses baculovirus-mediated^19^ expression of NMDAR, yielding rapid, titratable expression of NMDAR. The ability to titrate the amount of virus used for each subunit ensures optimal functional expression levels and prevents accumulation of homodimers in the cells. Furthermore, the assay is flexible for the combination of different subunits of NMDAR and does not require the time-consuming generation of stable cell lines. The use of weak ligand binding sites antagonist that compete with endogenous ligand in the media allows for the protection of the ligand binding site and, after washout of the antagonists, ensures sensitivity to exogenously added ligands to both, the glutamate and the glycine/D-serine binding site. To our knowledge, our approach describes the first high-throughput compatible assay for NMDAR with sensitivity to stimulation of both ligand binding sites and enables the study of thousands to millions of compounds at low costs.

The development of such an assay is timely given the advances in receptor pharmacology, the elucidation of its structure^28, 29^, and the genetic link of mutations in different subunits to disease^30^. Historically, targeting NMDARs has been challenging. Many antagonists failed clinical trials due to dose-limiting side effects, while agonists posed significant risk, as over-stimulation of this receptor is implicated in several pathologies^1, 2^. The discovery that different subunit compositions of the NMDAR heterotetrameric complex yield distinct receptor properties has led to the discovery of subunit-selective small molecules^31^. Targeting specific subunits may de-risk toxicities associated with non-selectivity over other receptor subtypes. In our approach, expression of NMDAR-subunits via baculovirus introduces a facile and time-effective way to study the effect of small molecules on multiple NMDAR subtype compositions. Our assay correctly recapitulated the selectivity of known NMDAR-inhibitors and is amenable to identify novel NMDAR-modulators with a desirable selectivity profile.

Elucidation of the NR1/NR2A receptor crystal structure has helped to understand the different mechanism of NMDAR modulators as well as to generate hypotheses on how to target and modulate different functional domains of the complex^32, 33^. For example, two distinct binding sites for allosteric modulators have been crystallographically described. A binding site for PAMs and NAMs is located at the heterodimer interface of the ligand-binding domains (NR1/NR2A), while a binding site for NAMs is located at the heterodimer interface of the amino-terminal domain of NR1/NR2B^34^. Another class of allosteric modulators is thought to bind at the amino-terminal domain of NR2^35, 36^. The use of mutants of NMDAR in our assay with mutations in known compound binding sites could facilitate the identification of small molecules with specific NMDAR binding sites, as exemplified in this study for mutants of the zinc-binding site or the binding site for GNE-8324.

In addition to subunit or binding site selectivity, an additional approach to target NMDAR receptors is to modulate the sensitivity to its co-agonists. Decreased D-serine levels are shown to be central in the development of schizophrenia^37, 38^. Our assay exhibits sensitivity to stimulation by glycine/D-serine and could facilitate the identification of small molecules that modulate ligand sensitivity of NMDAR or potentiate its function and hence mitigate the symptoms associated with receptor hypofunction^39, 40^.

As an alternative to targeting NMDAR directly, pharmacological modulation of proteins or signaling cascades that regulate NMDAR function and trafficking could present a promising path for drug discovery. Multiple publications have shown that various signaling molecules, including protein kinases, protein phosphatases, adaptor molecules and other intracellular signaling molecules can regulates NMDAR function and trafficking. For example, the cyclic AMP-protein kinase A (cAMP-PKA) has been shown to modulate the calcium permeability of NMDARs and to affect NMDAR-dependent long-term potentiation in synapses^41^. Src activity enhances activity of NMDAR and increases its interaction with the endocytotic signaling network leading to a longer retention time at the cell surface^42, 43^. Furthermore, protein kinase C, calcium/calmodulin-dependent kinase II and creatine kinase II have been associated with NMDAR function and trafficking^44–46^. We believe that our assay, combined with a genetic screening approach (like short interfering RNAs or CRISPR) can be used to identify novel regulators of NMDAR function in cells or to find compounds that modulate NMDAR activity indirectly as opposed to directly targeting NMDAR.

The ability to rapidly and in large scale study the biology of NMDARs, we believe, will enable the identification of novel therapeutics whose discovery has otherwise been impeded by the limitations of existing cell based approaches.

## Materials and Methods

### Cell culture

HEK293 cells were purchased from ATCC and cultured in DMEM supplemented with 10% fetal bovine serum at 37 °C, 5% CO_2_. HEK293-NR1-NR2A(teton) were obtained from Chantest (#CT6120) and were cultured following the manufacturer’s instructions.

### Compounds

Unless otherwise indicated, compounds were purchased from SigmaAldrich. MDL105,519, CGP070667, NVP-AAM077 and GN-8324 were synthesized in house. Compounds were dissolved in DMSO to a final concentration of 10 mM and stored at −20 °C. The library of 120 compounds was designed around the following NMDAR modulators: Ifenprodil^47^, CP101606^48^, RO25-6981^49^, MK801^50^, 7-CTKA^51^, TCN-201^20^, Spermine^52^, Sodium pregnenolone sulfate^53^, MDL105519^22^, NVP-AAM077^54^, CGP070667^23^, QNZ46^55^, CIQ^56^.

### Plasmids

A Gateway compatible BacMam vector with a strong CMV promoter was made by combining baculovirus vector elements from pFastBac1 (ThermoFischer, Cat #10360-014) with promoter elements from the Checkmate^TM^ pACT plasmid (Promega, Cat #C9341). The CMV-promotor replaced the late baculovirus polyhedron promotor from the vector. This strong promoter BacMam vector was Gateway adapted by cloning a Gateway® cassette from another vector into an XhoI site. The final vector was named pFastBac-CMV-GW-DEST. The full sequence of this vector can be found in the Supplementary Information. cDNAs for the GRIN1 (NM_007327), GRIN2A (NM_000833.3), GRIN2B (NM_000834.3) and Kir2.1 (NM_000891.2) proteins were purchased from GeneCopoeia as EZShuttle™ clones and cloned into the vector via an LR-reaction.

### Baculovirus Generation

For generation of recombinant baculovirus, the directions in the Invitrogen pFastBac manual were followed (Thermo Fisher cat #10712-024). The cell supernatants which contained the P1 viruses were filtered and added to 150 ml of Sf9 (Spodoptera frugiperda) insect cells at 2 × /ml in shaker flasks, and incubated at 27 °C at 100 rpm for 4–5 days to generate P2 viruses. P3 viruses were generated from P2 viruses using a 1:100 (v/v) dilution of the virus-containing supernatant to insect cells at 2 × 10^6^/ml.

### Cell viability assay

For measurement of cell viability, 10^4^ cells per 384-well were infected with baculovirus in suspension and seeded into a 384-well plate and treated with the indicated concentrations of inhibitor or DMSO for 16 h. Cell viability was assessed Cell Titer Glo (Promega) and measured on an Envision plate reader (PerkinElmer).

### Western blotting

Cell lysates were analyzed by SDS-PAGE and western blotting using standard protocols and as described in ref. 57 using the following primary antibodies: anti-NR1 (Cell Signaling #5704), anti-NR2A (Cell Signaling #4205), anti-Hsc70 (Epitomics #1776). Blots were analyzed using an Odyssey scanner (LICOR).

### Calcium flux assay

10^4^ cells per 384-well were infected with baculovirus in suspension, seeded into a clear bottom, poly-D-lysine treated 384-well plate (Corning) and treated with the indicated concentrations of protection compound (100 µM MDL105,519 or 5 µM CGP070667). 16 h after transduction, cell medium was replaced with calcium6-dye (Molecular Devices, R8192) in incubation buffer (HBSS pH 7.5, 20 mM HEPES, 1 mM MgCl_2_, 1 mM probenecid) and cells were incubated at 37 °C for 2 h. Before measurement, the dye-solution was replaced with 30 ul assay buffer (HBSS pH 7.5, 20 mM HEPES, 1.8 mM CaCl_2_). Cells were allowed to rest for 10 min at room temperature before measuring calcium flux on a FDSS7000 plate reader (Hamamatsu) with the following settings: 1 sec measurement interval, 200 ms exposure time, 2 × 2 binning, Ex480 nm:Em540 nm, sensitivity 1–3. In the beginning of each experiment baseline fluorescence (F0) was measure for 5–30 sec. For compound treatment, 10 µl of a 4× stock of compound diluted in assay buffer was added on the FDSS (speed 20 µl/s, tip height 1.8 mm) fluorescence was measured for 5 min (interval 1) before adding 10 µl of a 5× stock of ligand diluted in assay buffer and measurement of fluorescence for 5 min (interval 2). For single addition experiments, the maximal fluorescence ratio was calculated by dividing the maximal fluorescence in interval 1 by the average baseline fluorescence (F_max_/F_0_). For experiments requiring two additions, the integral of the fluorescence ratio was determined by calculating the area under the curve of the fluorescent ratios in interval 2. Data was analyzed as indicated and was normalized to controls located on the same plate.

### Data availability

The datasets generated during and/or analyzed during the current study are available from the corresponding author on reasonable request.

## Electronic supplementary material

Supplementary Information

## References

[1] Lau CG, Zukin RS (2007). NMDA receptor trafficking in synaptic plasticity and neuropsychiatric disorders. Nature reviews. Neuroscience.

[2] Traynelis SF (2010). Glutamate receptor ion channels: structure, regulation, and function. Pharmacol Rev.

[3] Marquard J (2015). Characterization of pancreatic NMDA receptors as possible drug targets for diabetes treatment. Nature medicine.

[4] Camilleri M, Di Lorenzo C (2012). Brain-gut axis: from basic understanding to treatment of IBS and related disorders. Journal of pediatric gastroenterology and nutrition.

[5] Nowak A (2014). The relationship of TP53 and GRIN2B gene polymorphisms with risk of occurrence and progression of primary open-angle glaucoma in a Polish population. Polish journal of pathology: official journal of the Polish Society of Pathologists.

[6] Farooq A (2014). Activation of N-methyl-d-aspartate receptor downregulates inflammasome activity and liver inflammation via a beta-arrestin-2 pathway. American journal of physiology. Gastrointestinal and liver physiology.

[7] Simma N (2014). NMDA-receptor antagonists block B-cell function but foster IL-10 production in BCR/CD40-activated B cells. Cell communication and signaling: CCS.

[8] Paoletti P, Bellone C, Zhou Q (2013). NMDA receptor subunit diversity: impact on receptor properties, synaptic plasticity and disease. Nature reviews. Neuroscience.

[9] Hunt DL, Castillo PE (2012). Synaptic plasticity of NMDA receptors: mechanisms and functional implications. Curr Opin Neurobiol.

[10] Huganir RL, Nicoll RA (2013). AMPARs and synaptic plasticity: the last 25 years. Neuron.

[11] Luscher, C. & Malenka, R. C. NMDA receptor-dependent long-term potentiation and long-term depression (LTP/LTD). *Cold Spring Harb Perspect Biol* **4**, 10.1101/cshperspect.a005710 (2012). 10.1101/cshperspect.a005710 PMC3367554 22510460

[12] Yashiro K, Philpot BD (2008). Regulation of NMDA receptor subunit expression and its implications for LTD, LTP, and metaplasticity. Neuropharmacology.

[13] Szydlowska K, Tymianski M (2010). Calcium, ischemia and excitotoxicity. Cell Calcium.

[14] Bettini E (2010). Identification and characterization of novel NMDA receptor antagonists selective for NR2A- over NR2B-containing receptors. J Pharmacol Exp Ther.

[15] Feuerbach D, Loetscher E, Neurdin S, Koller M (2010). Comparative pharmacology of the human NMDA-receptor subtypes R1-2A, R1-2B, R1-2C and R1-2D using an inducible expression system. Eur J Pharmacol.

[16] Hansen KB, Brauner-Osborne H, Egebjerg J (2008). Pharmacological characterization of ligands at recombinant NMDA receptor subtypes by electrophysiological recordings and intracellular calcium measurements. Comb Chem High Throughput Screen.

[17] Hansen KB (2010). Implementation of a fluorescence-based screening assay identifies histamine H3 receptor antagonists clobenpropit and iodophenpropit as subunit-selective N-methyl-D-aspartate receptor antagonists. J Pharmacol Exp Ther.

[18] Jambrina E (2016). An Integrated Approach for Screening and Identification of Positive Allosteric Modulators of N-Methyl-D-Aspartate Receptors. J Biomol Screen.

[19] Kost TA, Condreay JP, Jarvis DL (2005). Baculovirus as versatile vectors for protein expression in insect and mammalian cells. Nat Biotechnol.

[20] Hansen KB, Ogden KK, Traynelis SF (2012). Subunit-selective allosteric inhibition of glycine binding to NMDA receptors. J Neurosci.

[21] Morris RG, Anderson E, Lynch GS, Baudry M (1986). Selective impairment of learning and blockade of long-term potentiation by an N-methyl-D-aspartate receptor antagonist, AP5. Nature.

[22] Baron BM (1997). Pharmacological characterization of MDL 105,519, an NMDA receptor glycine site antagonist. Eur J Pharmacol.

[23] Auberson YP (2002). 5-Phosphonomethylquinoxalinediones as competitive NMDA receptor antagonists with a preference for the human 1A/2A, rather than 1A/2B receptor composition. Bioorg Med Chem Lett.

[24] Kim T (2004). The biochemical activation of T-type Ca^2+^ channels in HEK293 cells stably expressing alpha1G and Kir2.1 subunits. Biochem Biophys Res Commun.

[25] Horak M, Vlcek K, Chodounska H, Vyklicky L (2006). Subtype-dependence of N-methyl-D-aspartate receptor modulation by pregnenolone sulfate. Neuroscience.

[26] Hackos DH (2016). Positive Allosteric Modulators of GluN2A-Containing NMDARs with Distinct Modes of Action and Impacts on Circuit Function. Neuron.

[27] Fayyazuddin A, Villarroel A, Le Goff A, Lerma J, Neyton J (2000). Four residues of the extracellular N-terminal domain of the NR2A subunit control high-affinity Zn2+ binding to NMDA receptors. Neuron.

[28] Yi F (2016). Structural Basis for Negative Allosteric Modulation of GluN2A-Containing NMDA Receptors. Neuron.

[29] Romero-Hernandez A, Simorowski N, Karakas E, Furukawa H (2016). Molecular Basis for Subtype Specificity and High-Affinity Zinc Inhibition in the GluN1-GluN2A NMDA Receptor Amino-Terminal Domain. Neuron.

[30] Network & Pathway Analysis Subgroup of Psychiatric Genomics, C. Psychiatric genome-wide association study analyses implicate neuronal, immune and histone pathways. *Nat Neurosci* **18**, 199–209, 10.1038/nn.3922 (2015). 10.1038/nn.3922 PMC4378867 25599223

[31] Ogden KK, Traynelis SF (2011). New advances in NMDA receptor pharmacology. Trends in pharmacological sciences.

[32] Lee CH (2014). NMDA receptor structures reveal subunit arrangement and pore architecture. Nature.

[33] Karakas E, Furukawa H (2014). Crystal structure of a heterotetrameric NMDA receptor ion channel. Science.

[34] Zhu S, Paoletti P (2015). Allosteric modulators of NMDA receptors: multiple sites and mechanisms. Current opinion in pharmacology.

[35] Gielen M, Siegler Retchless B, Mony L, Johnson JW, Paoletti P (2009). Mechanism of differential control of NMDA receptor activity by NR2 subunits. Nature.

[36] Glasgow NG, Siegler Retchless B, Johnson JW (2015). Molecular bases of NMDA receptor subtype-dependent properties. The Journal of physiology.

[37] Balu DT, Coyle JT (2015). The NMDA receptor ‘glycine modulatory site’ in schizophrenia: D-serine, glycine, and beyond. Current opinion in pharmacology.

[38] Levin R (2015). Behavioral and cognitive effects of the N-methyl-D-aspartate receptor co-agonist D-serine in healthy humans: initial findings. Journal of psychiatric research.

[39] Moghaddam B, Javitt D (2012). From revolution to evolution: the glutamate hypothesis of schizophrenia and its implication for treatment. Neuropsychopharmacology: official publication of the American College of Neuropsychopharmacology.

[40] Hashimoto K, Malchow B, Falkai P, Schmitt A (2013). Glutamate modulators as potential therapeutic drugs in schizophrenia and affective disorders. European archives of psychiatry and clinical neuroscience.

[41] Skeberdis VA (2006). Protein kinase A regulates calcium permeability of NMDA receptors. Nat Neurosci.

[42] Salter MW, Kalia LV (2004). Src kinases: a hub for NMDA receptor regulation. Nature reviews. Neuroscience.

[43] Sans N (2003). NMDA receptor trafficking through an interaction between PDZ proteins and the exocyst complex. Nat Cell Biol.

[44] Chung HJ, Huang YH, Lau LF, Huganir RL (2004). Regulation of the NMDA receptor complex and trafficking by activity-dependent phosphorylation of the NR2B subunit PDZ ligand. J Neurosci.

[45] Strack S, McNeill RB, Colbran RJ (2000). Mechanism and regulation of calcium/calmodulin-dependent protein kinase II targeting to the NR2B subunit of the N-methyl-D-aspartate receptor. J Biol Chem.

[46] Lan JY (2001). Protein kinase C modulates NMDA receptor trafficking and gating. Nat Neurosci.

[47] Carter C, Rivy JP, Scatton B (1989). Ifenprodil and SL 82.0715 are antagonists at the polyamine site of the N-methyl-D-aspartate (NMDA) receptor. Eur J Pharmacol.

[48] Chenard BL (1995). 1S,2S)-1-(4-hydroxyphenyl)-2-(4-hydroxy-4-phenylpiperidino)-1-propanol: a potent new neuroprotectant which blocks N-methyl-D-aspartate responses. J Med Chem.

[49] Fischer G (1997). Ro 25-6981, a highly potent and selective blocker of N-methyl-D-aspartate receptors containing the NR2B subunit. Characterization *in vitro*. J Pharmacol Exp Ther.

[50] Lyle TA (1990). Structure and activity of hydrogenated derivatives of (+)-5-methyl-10,11-dihydro-5H-dibenzo[a,d]cyclohepten-5,10-imine (MK-801). J Med Chem.

[51] Leeson PD (1991). Kynurenic acid derivatives. Structure-activity relationships for excitatory amino acid antagonism and identification of potent and selective antagonists at the glycine site on the N-methyl-D-aspartate receptor. J Med Chem.

[52] Sacaan AI, Johnson KM (1989). Spermine enhances binding to the glycine site associated with the N-methyl-D-aspartate receptor complex. Mol Pharmacol.

[53] Malayev A, Gibbs TT, Farb DH (2002). Inhibition of the NMDA response by pregnenolone sulphate reveals subtype selective modulation of NMDA receptors by sulphated steroids. Br J Pharmacol.

[54] Feng B (2004). Structure-activity analysis of a novel NR2C/NR2D-preferring NMDA receptor antagonist: 1-(phenanthrene-2-carbonyl) piperazine-2,3-dicarboxylic acid. Br J Pharmacol.

[55] Hansen KB, Traynelis SF (2011). Structural and mechanistic determinants of a novel site for noncompetitive inhibition of GluN2D-containing NMDA receptors. J Neurosci.

[56] Mullasseril P (2010). A subunit-selective potentiator of NR2C- and NR2D-containing NMDA receptors. Nat Commun.

[57] Britschgi A (2013). Calcium-activated chloride channel ANO1 promotes breast cancer progression by activating EGFR and CAMK signaling. Proc Natl Acad Sci USA.

[58] Priestley T, Laughton P, Macaulay AJ, Hill RG, Kemp JA (1996). Electrophysiological characterisation of the antagonist properties of two novel NMDA receptor glycine site antagonists, L-695,902 and L-701,324. Neuropharmacology.

[59] Anis NA, Berry SC, Burton NR, Lodge D (1983). The dissociative anaesthetics, ketamine and phencyclidine, selectively reduce excitation of central mammalian neurones by N-methyl-aspartate. Br J Pharmacol.

