# Supplementary material for: A NMDA-receptor calcium influx assay sensitive to stimulation by glutamate and glycine/D-serine
Source: Sci Rep. 2017 Sep 14;7:11608. doi: 10.1038/s41598-017-11947-x (PMC5599507; doi:10.1038/s41598-017-11947-x)

A NMDA-receptor calcium influx assay sensitive to stimulation by glutamate and glycine/D-serine

Hongqiu Guo[1], L. Miguel Camargo[1], Fred Yeboah[1], Mary Ellen Digan[1], Honglin Niu[1], Yue Pan[2], Stephan Reiling[1], Gilberto Soler-Llavina[3], Wilhelm A. Weihofen[1], Hao-Ran Wang[1], Y. Gopi Shanker[3],Travis Stams[1], and Anke Bill*[1]

*Corresponding author: anke.bill@novartis.com

Affiliations:

[1] Novartis Institutes of Biomedical Research, Chemical Biology and Therapeutics, 250 Massachusetts Avenue, Cambridge, MA 02139.

[2] Novartis Institutes of Biomedical Research, Global Discovery Chemistry, 181 Massachusetts Avenue, Cambridge, MA 02139.

[3] Novartis Institutes of Biomedical Research, Neuroscience, 181 Massachusetts Avenue, Cambridge, MA 02139.

Supplementary Figure 1

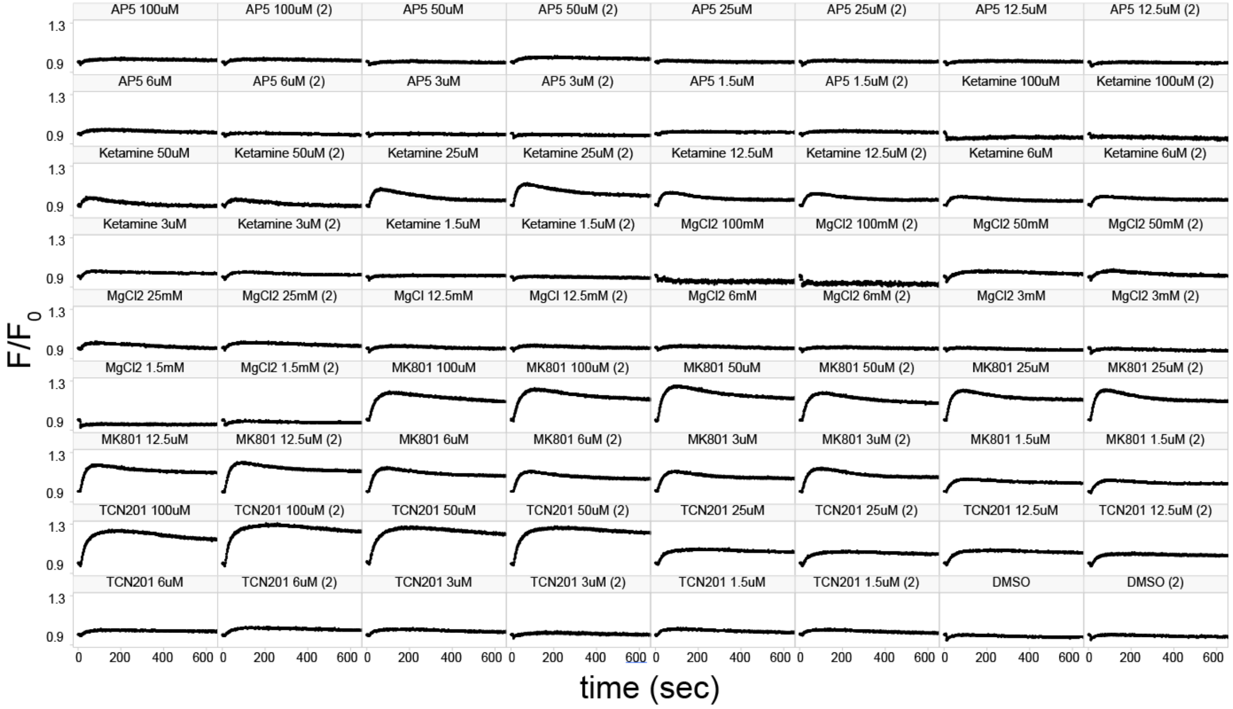

Supplementary Figure 1 Protection of NMDAR-expressing cells with MDL105,519, but not MK801 or Ketamine, facilitates NMDAR-activity in functional assay

NMDAR-mediated calcium flux in HEK293 cells transduced with baculovirus. NR1/2A-transduced HEK293 cells were treated with the indicated compounds. 16h later, cells were washed three times, loaded with Calcium6 dye and NMDAR-mediated calcium flux was measured after stimulation with 100 µM glycine/glutamate. Data represents the raw traces of the fluorescence ratio (fluorescence/baseline fluorescence, F/F0) of one representative experiment. The summary of the data across multiple experiments is shown in Figure 2A.

Supplementary Figure 2

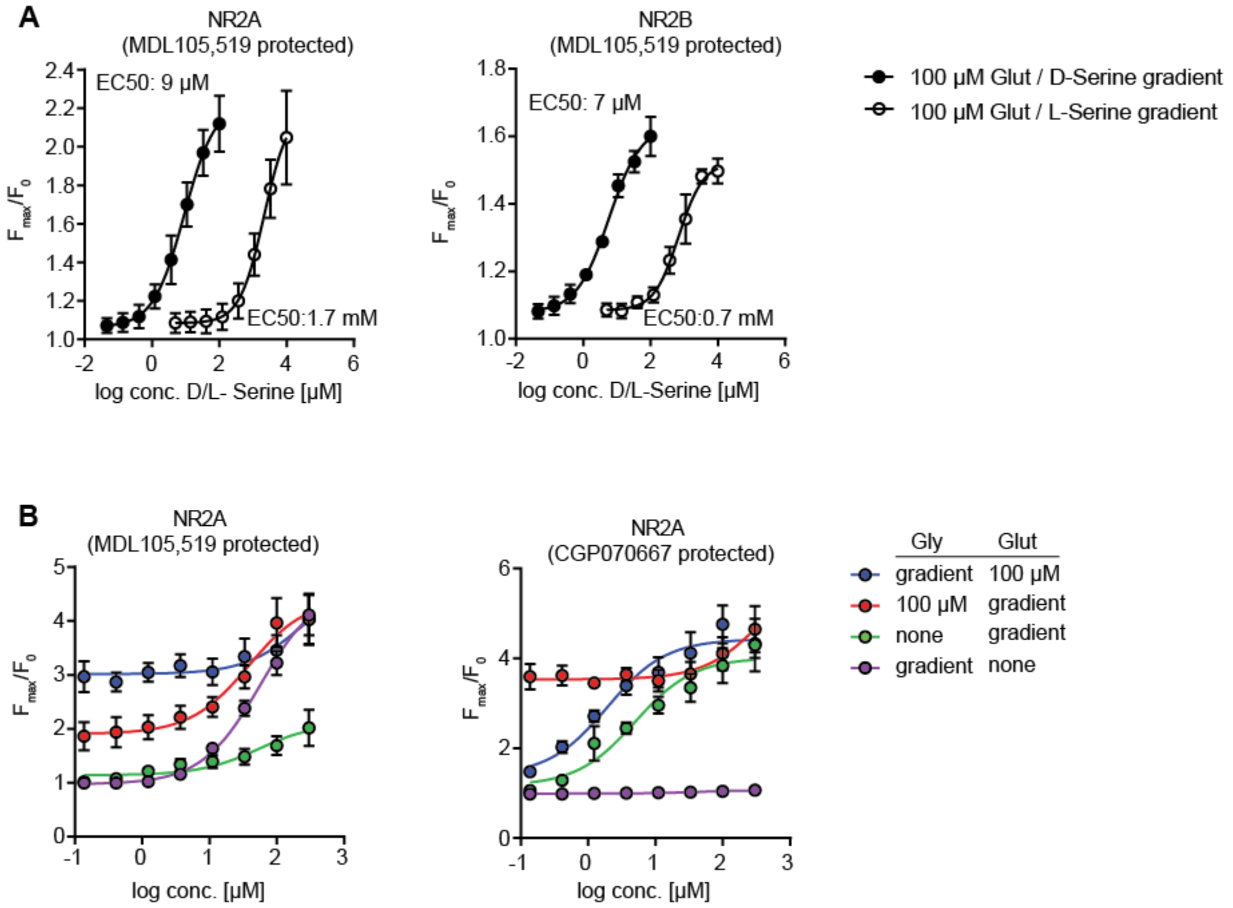

Supplementary Figure 2 Baculovirus mediated expression of NMDAR recapitulates expected NMDAR biology

(A) Sensitivity of NMDAR-mediated calcium flux to D-serine and L-serine. HEK293 cells were transduced with baculovirus encoding NR1 and NR2A or NR2B in the presence of MDL105,519 and NMDAR-mediated calcium flux was measured as described in the presence of varying amounts of D-serine or L-serine and saturating concentration of glutamate. Data represents the mean of the maximal fluorescence ratio ± SEM of four experiments.

(B) HEK293 cells stably expressing NR1 and NR2A under the control of a tetracycline-inducible promotor were seeded and induced with tetracycline in the presence of MDL105.519 or CGP070667, and NMDAR-mediated calcium flux was measured as previously described after addition of the indicated concentration of glycine and glutamate. Data represents the mean of the maximal fluorescence ratio ± SEM of three experiments.

Supplementary Figure 3

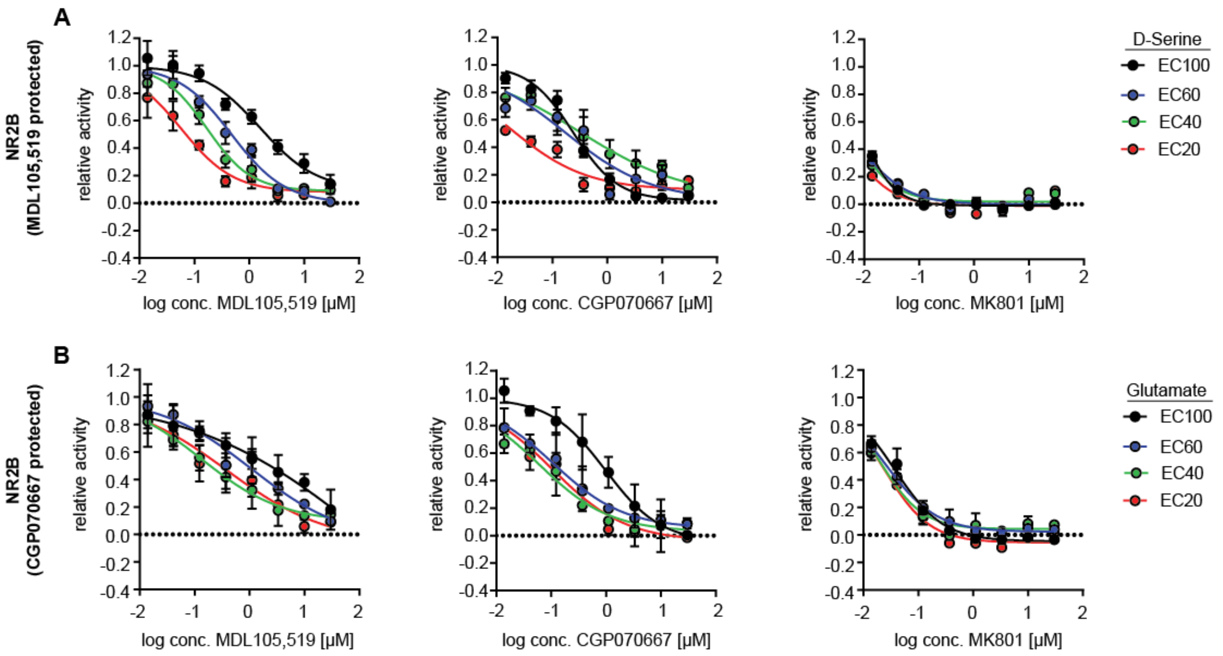

Supplementary Figure 3 Characterization of the mode of action of NMDAR (NR2B) inhibitors

(A-B) Sensitivity of NR1/2B mediated calcium flux to inhibition by MDL105,519 (first column), CGP070667 (middle column) or MK801 (last column) at different ligand concentrations. HEK293 cells were transduced with baculovirus encoding NR1 and NR2B in the presence of MDL105,519 (A) or CGP070667 (B), and NMDAR-mediated calcium flux was measured as previously described in the presence of the indicated ligand concentrations. The integral of the maximal fluorescence ratio was normalized to the activity measured in the absence of inhibitor (DMSO only) at the respective ligand concentration. Data represents the mean ± SEM of three experiments.

Sequence of pFastBac1-CMV/GW-DEST

TCGAGGGTGGTGGTGGTAGTGGTGGTGGTGGTAGTGGATCAACAAGTTTGTACAAAAAAGCTGAACGAGA
AACGTAAAATGATATAAATATCAATATATTAAATTAGATTTTGCATAAAAAACAGACTACATAATACTGT
AAAACACAACATATCCAGTCACTATGGCGGCCGCATTAGGCACCCCAGGCTTTACACTTTATGCTTCCGG
CTCGTATAATGTGTGGATTTTGAGTTAGGATCCGGCGAGATTTTCAGGAGCTAAGGAAGCTAAAATGGAG
AAAAAAATCACTGGATATACCACCGTTGATATATCCCAATGGCATCGTAAAGAACATTTTGAGGCATTTC
AGTCAGTTGCTCAATGTACCTATAACCAGACCGTTCAGCTGGATATTACGGCCTTTTTAAAGACCGTAAA
GAAAAATAAGCACAAGTTTTATCCGGCCTTTATTCACATTCTTGCCCGCCTGATGAATGCTCATCCGGAA
TTCCGTATGGCAATGAAAGACGGTGAGCTGGTGATATGGGATAGTGTTCACCCTTGTTACACCGTTTTCC
ATGAGCAAACTGAAACGTTTTCATCGCTCTGGAGTGAATACCACGACGATTTCCGGCAGTTTCTACACAT
ATATTCGCAAGATGTGGCGTGTTACGGTGAAAACCTGGCCTATTTCCCTAAAGGGTTTATTGAGAATATG
TTTTTCGTCTCAGCCAATCCCTGGGTGAGTTTCACCAGTTTTGATTTAAACGTGGCCAATATGGACAACT
TCTTCGCCCCCGTTTTCACCATGGGCAAATATTATACGCAAGGCGACAAGGTGCTGATGCCGCTGGCGAT
TCAGGTTCATCATGCCGTCTGTGATGGCTTCCATGTCGGCAGAATGCTTAATGAATTACAACAGTACTGC
GATGAGTGGCAGGGCGGGGCGTAAAGATCTGGATCCGGCTTACTAAAAGCCAGATAACAGTATGCGTATT
TGCGCGCTGATTTTTGCGGTATAAGAATATATACTGATATGTATACCCGAAGTATGTCAAAAAGAGGTGT
GCTATGAAGCAGCGTATTACAGTGACAGTTGACAGCGACAGCTATCAGTTGCTCAAGGCATATATGATGT
CAATATCTCCGGTCTGGTAAGCACAACCATGCAGAATGAAGCCCGTCGTCTGCGTGCCGAACGCTGGAAA
GCGGAAAATCAGGAAGGGATGGCTGAGGTCGCCCGGTTTATTGAAATGAACGGCTCTTTTGCTGACGAGA
ACAGGGACTGGTGAAATGCAGTTTAAGGTTTACACCTATAAAAGAGAGAGCCGTTATCGTCTGTTTGTGG
ATGTACAGAGTGATATTATTGACACGCCCGGGCGACGGATGGTGATCCCCCTGGCCAGTGCACGTCTGCT
GTCAGATAAAGTCTCCCGTGAACTTTACCCGGTGGTGCATATCGGGGATGAAAGCTGGCGCATGATGACC
ACCGATATGGCCAGTGTGCCGGTCTCCGTTATCGGGGAAGAAGTGGCTGATCTCAGCCACCGCGAAAATG
ACATCAAAAACGCCATTAACCTGATGTTCTGGGGAATATAAATGTCAGGCTCCCTTATACACAGCCAGTC
TGCAGGTCGACCATAGTGACTGGATATGTTGTGTTTTACAGTATTATGTAGTCTGTTTTTTATGCAAAAT
CTAATTTAATATATTGATATTTATATCATTTTACGTTTCTCGTTCAGCTTTCTTGTACAAAGTGGTGGGC
CGCACAGCTGTATACACGTGCAAGCCAGCCAGAACTCGCCCCGGAAGACCCCGAGGATCTCGAGGCATGC
GGTACCAAGCTTGTCGAGAAGTACTAGAGGATCATAATCAGCCATACCACATTTGTAGAGGTTTTACTTG
CTTTAAAAAACCTCCCACACCTCCCCCTGAACCTGAAACATAAAATGAATGCAATTGTTGTTGTTAACTT
GTTTATTGCAGCTTATAATGGTTACAAATAAAGCAATAGCATCACAAATTTCACAAATAAAGCATTTTTT
TCACTGCATTCTAGTTGTGGTTTGTCCAAACTCATCAATGTATCTTATCATGTCTGGATCTGATCACTGC
TTGAGCCTAGGAGATCCGAACCAGATAAGTGAAATCTAGTTCCAAACTATTTTGTCATTTTTAATTTTCG
TATTAGCTTACGACGCTACACCCAGTTCCCATCTATTTTGTCACTCTTCCCTAAATAATCCTTAAAAACT
CCATTTCCACCCCTCCCAGTTCCCAACTATTTTGTCCGCCCACAGCGGGGCATTTTTCTTCCTGTTATGT
TTTTAATCAAACATCCTGCCAACTCCATGTGACAAACCGTCATCTTCGGCTACTTTTTCTCTGTCACAGA
ATGAAAATTTTTCTGTCATCTCTTCGTTATTAATGTTTGTAATTGACTGAATATCAACGCTTATTTGCAG
CCTGAATGGCGAATGGGACGCGCCCTGTAGCGGCGCATTAAGCGCGGCGGGTGTGGTGGTTACGCGCAGC
GTGACCGCTACACTTGCCAGCGCCCTAGCGCCCGCTCCTTTCGCTTTCTTCCCTTCCTTTCTCGCCACGT
TCGCCGGCTTTCCCCGTCAAGCTCTAAATCGGGGGCTCCCTTTAGGGTTCCGATTTAGTGCTTTACGGCA
CCTCGACCCCAAAAAACTTGATTAGGGTGATGGTTCACGTAGTGGGCCATCGCCCTGATAGACGGTTTTT
CGCCCTTTGACGTTGGAGTCCACGTTCTTTAATAGTGGACTCTTGTTCCAAACTGGAACAACACTCAACC
CTATCTCGGTCTATTCTTTTGATTTATAAGGGATTTTGCCGATTTCGGCCTATTGGTTAAAAAATGAGCT
GATTTAACAAAAATTTAACGCGAATTTTAACAAAATATTAACGTTTACAATTTCAGGTGGCACTTTTCGG
GGAAATGTGCGCGGAACCCCTATTTGTTTATTTTTCTAAATACATTCAAATATGTATCCGCTCATGAGAC
AATAACCCTGATAAATGCTTCAATAATATTGAAAAAGGAAGAGTATGAGTATTCAACATTTCCGTGTCGC
CCTTATTCCCTTTTTTGCGGCATTTTGCCTTCCTGTTTTTGCTCACCCAGAAACGCTGGTGAAAGTAAAA
GATGCTGAAGATCAGTTGGGTGCACGAGTGGGTTACATCGAACTGGATCTCAACAGCGGTAAGATCCTTG

AGAGTTTTCGCCCCGAAGAACGTTTTCCAATGATGAGCACTTTTAAAGTTCTGCTATGTGGCGCGGTATT
ATCCCGTATTGACGCCGGGCAAGAGCAACTCGGTCGCCGCATACACTATTCTCAGAATGACTTGGTTGAG
TACTCACCAGTCACAGAAAAGCATCTTACGGATGGCATGACAGTAAGAGAATTATGCAGTGCTGCCATAA
CCATGAGTGATAACACTGCGGCCAACTTACTTCTGACAACGATCGGAGGACCGAAGGAGCTAACCGCTTT
TTTGCACAACATGGGGGATCATGTAACTCGCCTTGATCGTTGGGAACCGGAGCTGAATGAAGCCATACCA
AACGACGAGCGTGACACCACGATGCCTGTAGCAATGGCAACAACGTTGCGCAAACTATTAACTGGCGAAC
TACTTACTCTAGCTTCCCGGCAACAATTAATAGACTGGATGGAGGCGGATAAAGTTGCAGGACCACTTCT
GCGCTCGGCCCTTCCGGCTGGCTGGTTTATTGCTGATAAATCTGGAGCCGGTGAGCGTGGGTCTCGCGGT
ATCATTGCAGCACTGGGGCCAGATGGTAAGCCCTCCCGTATCGTAGTTATCTACACGACGGGGAGTCAGG
CAACTATGGATGAACGAAATAGACAGATCGCTGAGATAGGTGCCTCACTGATTAAGCATTGGTAACTGTC
AGACCAAGTTTACTCATATATACTTTAGATTGATTTAAAACTTCATTTTTAATTTAAAAGGATCTAGGTG
AAGATCCTTTTTGATAATCTCATGACCAAAATCCCTTAACGTGAGTTTTCGTTCCACTGAGCGTCAGACC
CCGTAGAAAAGATCAAAGGATCTTCTTGAGATCCTTTTTTTCTGCGCGTAATCTGCTGCTTGCAAACAAA
AAAACCACCGCTACCAGCGGTGGTTTGTTTGCCGGATCAAGAGCTACCAACTCTTTTTCCGAAGGTAACT
GGCTTCAGCAGAGCGCAGATACCAAATACTGTCCTTCTAGTGTAGCCGTAGTTAGGCCACCACTTCAAGA
ACTCTGTAGCACCGCCTACATACCTCGCTCTGCTAATCCTGTTACCAGTGGCTGCTGCCAGTGGCGATAA
GTCGTGTCTTACCGGGTTGGACTCAAGACGATAGTTACCGGATAAGGCGCAGCGGTCGGGCTGAACGGGG
GGTTCGTGCACACAGCCCAGCTTGGAGCGAACGACCTACACCGAACTGAGATACCTACAGCGTGAGCATT
GAGAAAGCGCCACGCTTCCCGAAGGGAGAAAGGCGGACAGGTATCCGGTAAGCGGCAGGGTCGGAACAGG
AGAGCGCACGAGGGAGCTTCCAGGGGGAAACGCCTGGTATCTTTATAGTCCTGTCGGGTTTCGCCACCTC
TGACTTGAGCGTCGATTTTTGTGATGCTCGTCAGGGGGGCGGAGCCTATGGAAAAACGCCAGCAACGCGG
CCTTTTTACGGTTCCTGGCCTTTTGCTGGCCTTTTGCTCACATGTTCTTTCCTGCGTTATCCCCTGATTC
TGTGGATAACCGTATTACCGCCTTTGAGTGAGCTGATACCGCTCGCCGCAGCCGAACGACCGAGCGCAGC
GAGTCAGTGAGCGAGGAAGCGGAAGAGCGCCTGATGCGGTATTTTCTCCTTACGCATCTGTGCGGTATTT
CACACCGCAGACCAGCCGCGTAACCTGGCAAAATCGGTTACGGTTGAGTAATAAATGGATGCCCTGCGTA
AGCGGGTGTGGGCGGACAATAAAGTCTTAAACTGAACAAAATAGATCTAAACTATGACAATAAAGTCTTA
AACTAGACAGAATAGTTGTAAACTGAAATCAGTCCAGTTATGCTGTGAAAAAGCATACTGGACTTTTGTT
ATGGCTAAAGCAAACTCTTCATTTTCTGAAGTGCAAATTGCCCGTCGTATTAAAGAGGGGCGTGGCCAAG
GGCATGGTAAAGACTATATTCGCGGCGTTGTGACAATTTACCGAACAACTCCGCGGCCGGGAAGCCGATC
TCGGCTTGAACGAATTGTTAGGTGGCGGTACTTGGGTCGATATCAAAGTGCATCACTTCTTCCCGTATGC
CCAACTTTGTATAGAGAGCCACTGCGGGATCGTCACCGTAATCTGCTTGCACGTAGATCACATAAGCACC
AAGCGCGTTGGCCTCATGCTTGAGGAGATTGATGAGCGCGGTGGCAATGCCCTGCCTCCGGTGCTCGCCG
GAGACTGCGAGATCATAGATATAGATCTCACTACGCGGCTGCTCAAACCTGGGCAGAACGTAAGCCGCGA
GAGCGCCAACAACCGCTTCTTGGTCGAAGGCAGCAAGCGCGATGAATGTCTTACTACGGAGCAAGTTCCC
GAGGTAATCGGAGTCCGGCTGATGTTGGGAGTAGGTGGCTACGTCTCCGAACTCACGACCGAAAAGATCA
AGAGCAGCCCGCATGGATTTGACTTGGTCAGGGCCGAGCCTACATGTGCGAATGATGCCCATACTTGAGC
CACCTAACTTTGTTTTAGGGCGACTGCCCTGCTGCGTAACATCGTTGCTGCTGCGTAACATCGTTGCTGC
TCCATAACATCAAACATCGACCCACGGCGTAACGCGCTTGCTGCTTGGATGCCCGAGGCATAGACTGTAC
AAAAAAACAGTCATAACAAGCCATGAAAACCGCCACTGCGCCGTTACCACCGCTGCGTTCGGTCAAGGTT
CTGGACCAGTTGCGTGAGCGCATACGCTACTTGCATTACAGTTTACGAACCGAACAGGCTTATGTCAACT
GGGTTCGTGCCTTCATCCGTTTCCACGGTGTGCGTCACCCGGCAACCTTGGGCAGCAGCGAAGTCGAGGC
ATTTCTGTCCTGGCTGGCGAACGAGCGCAAGGTTTCGGTCTCCACGCATCGTCAGGCATTGGCGGCCTTG
CTGTTCTTCTACGGCAAGGTGCTGTGCACGGATCTGCCCTGGCTTCAGGAGATCGGAAGACCTCGGCCGT
CGCGGCGCTTGCCGGTGGTGCTGACCCCGGATGAAGTGGTTCGCATCCTCGGTTTTCTGGAAGGCGAGCA
TCGTTTGTTCGCCCAGGACTCTAGCTATAGTTCTAGTGGTTGGCTACCCTACGTCGACGAGCTCACTAGT
TCAATATTGGCCATTAGCCATATTATTCATTGGTTATATAGCATAAATCAATATTGGCTATTGGCCATTG
CATACGTTGTATCTATATCATAATATGTACATTTATATTGGCTCATGTCCAATATGACCGCCATGTTGGC
ATTGATTATTGACTAGTTATTAATAGTAATCAATTACGGGGTCATTAGTTCATAGCCCATATATGGAGTT

CCGCGTTACATAACTTACGGTAAATGGCCCGCCTGGCTGACCGCCCAACGACCCCCGCCCATTGACGTCA
ATAATGACGTATGTTCCCATAGTAACGCCAATAGGGACTTTCCATTGACGTCAATGGGTGGAGTATTTAC
GGTAAACTGCCCACTTGGCAGTACATCAAGTGTATCATATGCCAAGTCCGCCCCCTATTGACGTCAATGA
CGGTAAATGGCCCGCCTGGCATTATGCCCAGTACATGACCTTACGGGACTTTCCTACTTGGCAGTACATC
TACGTATTAGTCATCGCTATTACCATGGTGATGCGGTTTTGGCAGTACACCAATGGGCGTGGATAGCGGT
TTGACTCACGGGGATTTCCAAGTCTCCACCCCATTGACGTCAATGGGAGTTTGTTTTGGCACCAAAATCA
ACGGGACTTTCCAAAATGTCGTAACAACTGCGATCGCCCGCCCCGTTGACGCAAATGGGCGGTAGGCGTG
TACGGTGGGAGGTCTATATAAGCAGAGCTCGTTTAGTGAACCGTCAGATCACTAGAAGCTTTATTGCGGT
AGTTTATCACAGTTAAATTGCTAACGCAGTCAGTGCTTCTGACACAACAGTCTCGAACTTAAGCTGCAGT
GACTCTCTTAAGGTAGCCTTGCAGAAGTTGGTCGTGAGGCACTGGGCAGGTAAGTATCAAGGTTACAAGA
CAGGTTTAAGGAGACCAATAGAAACTGGGCTTGTCGAGACAGAGAAGACTCTTGCGTTTCTGATAGGCAC
CTATTGGTCTTACTGACATCCACTTTGCCTTTCTCTCCACAGGTGTCCACTCCCAGTTCAATTACAGCTC
TTAAGGCTAGAGTACTTAATACGACTCACTATAGGCTAGCCAGCTTGAAGCAAGCCTCCC

Western blot images

NR1 (full blot image)

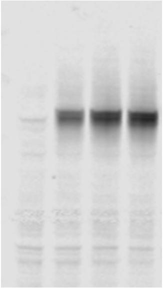

Hsc70 loading control for NR1 (full blot image, not shown in figure)

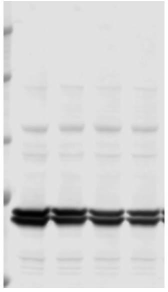

NR2A (full blot image)

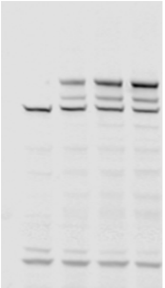

Hsc70 loading control for NR2A (full blot image)

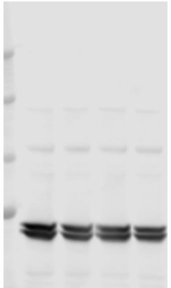

Supplement: Supplementary file 1 — Supplementary Information [file 41598_2017_11947_MOESM1_ESM.pdf]
